# Detection and description of a novel *Psychrobacter glacincola* infection in some Red Sea marine fishes in Hurghada, Egypt

**DOI:** 10.1186/s12917-022-03542-8

**Published:** 2023-01-30

**Authors:** Mohamed Raafat El-Sayed, Arafah M. Emam, Ahmed Elsayed Osman, Mohamed Abd El-Aziz Ahmed Abd El-Galil, Haitham Helmy Sayed

**Affiliations:** 1 Department of Fish Diseases and Management, Faculty of Veterinary Medicine, Sohag University, Sohag, Egypt; 2 National Institute of Oceanography and Fisheries, NIOF, Cairo, Egypt; 3 Department of Biochemistry, Faculty of Veterinary Medicine, Sohag University, Sohag, Egypt; 4 Department of Microbiology, Faculty of Veterinary Medicine, Sohag University, Sohag, Egypt

**Keywords:** Psychrobacter, Glacincola, Red Sea, Fish, Hurghada

## Abstract

An important food-producing sector in Egypt is aquaculture and fisheries; however, several pathogenic microorganisms lead to high mortalities and significant economic losses. The occurrence of *Psychrobacter glacincola* infection among 180 wild marine fishes collected from the Red sea at Hurghada, Egypt were investigated in the present study. The disease prevalence rate was 6.7%. The recovered isolates were subjected to biochemical and molecular identification. The study also investigated pathogenicity and the antibiogram profile of the recovered isolates. The clinical examination of the infected fish revealed various signs that included lethargy and sluggish movement, hemorrhages and ulcers on the body and the operculum, scale loss, and fin congestion and rot, especially at the tail fin. Furthermore, during postmortem examination, congestion of the liver, spleen, and kidney was observed. Interestingly, 12 isolates were recovered and were homogenous bacteriologically and biochemically. The phylogenetic analysis based on 16S rRNA gene confirmed that MRB62 identified strain was closely related the genus Psychrobacter and identified as *P. glacincola* and was pathogenic to *Rhabdosargus haffara* fish, causing 23.3% mortality combined with reporting a series of clinical signs similar to that found in naturally infected fishes. The present study also showed that *P. glacincola* isolates were sensitive to all antibiotics used for sensitivity testing. Our findings add to the body of knowledge regarding the occurrence of pathogenic *P. glacincola* infection in Egyptian marine fishes and its potential effects on fish. Future large-scale surveys exploring this bacterium among other freshwater and marine fishes in Egypt would be helpful for the implementation of effective strategies for the prevention and control of this infection are warranted.

**Supplementary Information:**

The online version contains supplementary material available at 10.1186/s12917-022-03542-8.

## Introduction

An important source of income in many developing countries is marine fish, besides being one of the major investment choices for national fishermen [1]. Egypt is bordered by the Red Sea on the east, which is 2250 km in length with an average depth of 490 m. Importantly, the Red Sea has a unique composition of fish species, which consists of 1166 species. Some of the most economically important Red Sea marine fish species in Egypt include marbled spinefoot (*Siganus rivulatus*), blackspot snapper (*Lutjanus ehrenbergii)*, snubnose emperor (*Lethrinus borbonicus*), blue-barred parrotfish (*Scarus ghobban*), Haffara seabream (*Rhabdosargus haffara*), and broomtail wrasse (*Cheilinus lunulatus*). However, this important sector is challenged by a wide range of serious pathogenic organisms, affecting diverse marine fishes and shellfish [2]. Among others, bacteria are the most prevalent cause of morbidity and mortality among wild populations of fish, resulting in major economic losses in this sector [3]. Generally, several bacterial strains are normally present in aquatic environments and their simple presence in marine environments is insufficient to cause a disease outbreak; however, they might become highly pathogenic under stressful conditions [4].

Among others, *Psychrobacter* species have been isolated from various marine environments and seawater, which is considered a good habitat for these species [5]. The presence of *Psychrobacter* spp. was also significantly associated with other environmental factors, that is, temperature, neutral pH, high salinity, higher concentrations of potassium and magnesium [6], and hydrocarbon-contaminated aquatic environments [7, 8]. Furthermore, members of the genus *Psychrobacter* have been isolated from the gastrointestinal tract, skin, and gills of apparently healthy Atlantic salmon [9], Atlantic cod [10], juvenile grouper [11], and Atlantic mackerel [12]. To enhance the growth rate and immune system efficiency in fish, *Psychrobacter* spp. have been used as probiotics [13] since they induce growth inhibition of some pathogenic bacteria and enhance the growth of many beneficial or neutral bacteria in the gut of fish [14]. For some fish species, some *Psychrobacter* strains were recorded as opportunistic pathogenic microorganisms. Some previous studies [15, 16] reported *P. immobilis* infection in rainbow trout (*Oncorhynchus mykiss*) and Atlantic salmon. *Psychrobacter* isolates such as *P. immobilis* and *P. phenylpyruvicus* have been isolated in clinical samples from brain tissue, urine, ears, wounds, cerebrospinal fluid, and blood and have been reported to be opportunistic pathogens in humans [17–21]. Another species named *P. glacincola* was isolated as a novel strain from sea ice cores in Antarctica [22], mud of the Shetland Islands [23], processed fresh edible sea urchin in Tokyo [24], red tanner crab [16], water of aquaculture and agriculture run off of Ria de Aveiro [25], and sediment samples of King George Island, Antarctica [26].

Revising the available literature, the prevalence of bacterial diseases was documented in several cultured and wild freshwater fish species from Egypt. However, only a few bacteriological surveys were performed on marine fish species to track disease outbreaks. More importantly, no available data was reported regarding the *P. glacincola* infection among fishes worldwide. Given the above information, the present study investigated the occurrence of *P. glacincola* among some fish species inhibited by the Red Sea in Hurghada, Egypt, through clinical examination, bacteriological isolation, phenotyping, and biochemical and molecular identification of the isolated strains. Combined with antimicrobial susceptibility testing of the recovered *P. glacincola* isolates, the study also included a pathogenicity test for pathogenicity verification of the isolated strains.

## Material and methods

### Study area and sampling

From October 2019 to March 2020, a total of 180 fish were obtained from Hurghada, Red Sea governorate, Egypt. The examined fish species were as follows: snubnose emperor (*Lethrinus borbonicus*), blackspot snapper (*Lutjanus ehrenbergii*), marbled spinefoot (*Siganus rivulatus*), Haffara seabream (*Rhabdosargus haffara*), blue-barred parrotfish (*Scarus ghobban*), and broomtail wrasse (*Cheilinus lunulatus*). The rate of sampling was 30 fish from each species. Fish were collected and transported immediately to the indoor aquarium at the National Institute of Oceanography and Fisheries in Hurghada, Egypt, for clinical and bacteriological examinations. We confirm that all experimental methods were performed in accordance with the relevant guidelines and regulations of the National Institute of Oceanography and Fisheries Committee for ethical care of marine organisms and experimental animals (NIOF-AICUC).

### Clinical and postmortem examination

Clinical and postmortem examinations of fish were conducted to detect external and internal clinical abnormalities according to the method described by Schäperclaus [27]. Fishes were anesthetized with tricaine methanesulfonate (MS222-Sigma-Aldrich) prior to examination.

### Bacterial isolation

Under completely aseptic conditions, bacteriological samples from the liver, kidney, and spleen were collected by sterile loop. The collected samples were then inoculated into brain heart infusion Broth supplemented with 1.5% NaCl (Oxoid, England) and incubated aerobically at 22 °C for up to 48 h, then streaked onto tryptone soy agar (Oxoid, England) supplemented with 1.5% NaCl and incubated at 22 °C for up to 48 h [28]. The recovered isolates were then preserved at − 80 °C in tryptone soy broth supplemented with 25% glycerol for further identification.

### Phenotyping and biochemical identification of the isolated bacteria

The suspected isolates were identified through their morphological characteristics, Gram staining, motility test, oxidase test, and API 20E system (BioMerieux, France) according to the manufacturer’s instructions.

### Molecular identification of the recovered isolates and sequence analysis

Using the GeneJET genomic DNA purification kit (Thermo Fisher Scientific, EU), the bacterial DNA was extracted from the recovered isolates according to the manufacturer’s instructions. The extracted DNA was then stored at − 20 °C until use. Later, to amplify the hypervariable segment of 16S rRNA using a set of universal primers, polymerase chain reaction (PCR) was conducted [29], which is shown in Table 1. PCR was conducted in 50-μL mixtures, according to the Master Mix manufacturer’s instructions, which contained 25-μL of Master Mix, 2 μL of each primer, 4 μL of the extracted DNA, and nuclease-free water up to 50 μL. As shown in Table 1, PCR was conducted in a thermocycler (Applied Biosystems, USA) under the conditions previously described [30]. In brief, the initial denaturation was performed at 95 °C for 5 min, followed by 35 cycles of denaturation at 94 °C for 1 min, annealing at 55 °C for 1 min, and extension at 72 °C for 1.5 min, followed by a final extension step at 72 °C for 10 min. The amplicons (1500 base pairs) were purified and sequenced by the 3500 Genetic Analyzer (Applied Biosystems, USA). The draft genome sequence of strain MR-B62 was sequenced at SolGent Co., Ltd. Bio-Industry development site (South Korea) using Sanger dideoxy sequencing technology. The sequences of the recovered isolates were analyzed using MEGA 7.0 software and compared to those available in the GenBank database. Using the maximum composite likelihood method, evolutionary distances were computed. A phylogenetic tree based on the 16S rRNA gene sequences was reconstructed by the neighbor-joining method [31].

**Table 1:** Primer sets used in molecular characterization of *P. glacincola* isolates

Genes	Primers sequence (5*'*‐3*'*)	PCR conditions	Product sizes/ (bp)
16S rRNA	F27 AGAGTTTGATCMTGGCTCAGTTGTCCGGGTTGTACTCGTC 1492R GGTTACCTTGTTACGACTT	Initial denaturation 95 °C /5 min.35 cycles of denaturation at 94 °C/60 s Annealing at 55°C/1min extension at 72°C/1.5 min	1500

### Pathogenicity testing

A total of 40 acclimated healthy Haffara seabream (*Rhabdosargus haffara*) fish with an average body weight 50 ± 5 g were obtained from the National Institute of Oceanography and Fisheries, Hurghada, Egypt. These fish were experimentally infected with the bacterial suspension (*P. glacincola*) recovered from naturally infected marine fish. Fish were divided into four equal groups; three groups were intraperitoneally (IP) injected with *P. glacincola* suspension at a dose of 0.1 mL (3 × 10^7^ CFU) [32], and the fish of the 4^th^ group were IP injected with 0.1 mL of sterile saline and used as a control. Fish were closely observed daily for 2 weeks, and the clinical signs and mortalities were recorded. Freshly dead fish were subjected to postmortem examination, bacteriological isolation, and identification of *P. glacincola* from the liver, spleen, and kidney.

### Antimicrobial susceptibility test of the recovered *P. glacincola* isolates

Using the Kirby Bauer disc diffusion method, the antimicrobial susceptibility of the recovered isolates was determined. The following antibiotic discs were used: tetracycline (30 µg), ciprofloxacin (5 µg), ofloxacin (5 µg), oxolinic acid (2 µg), erythromycin (15 µg), chloramphenicol (30 µg), amoxicillin/clavulanic acid (30 µg), cephalothin (30 µg), amikacin (30 µg), streptomycin (10 µg), cefotaxime (30 µg), trimethoprim/sulfamethoxazole (25 µg), gentamycin (10 µg), clindamycin (2 µg), flucloxacillin (5 µg), and tobramycin (5 µg). The recovered isolates were streaked into Mueller–Hinton agar (Oxoid, England), the antibiotic discs were placed, and the inoculated plate was incubated at 25 °C for 48 h. Diameters of the inhibition zones were measured and interpreted according to the Clinical and Laboratory Standards Institute (2012) (33).

### Statistical analysis

The Kaplan–Meier curve was used to calculate the infected and control trials' survival rates over time. The number of fish that survived is divided by the number of dead samples.

## Results

### Clinical signs of *P. glacincola* infection

**Fig. 1:**
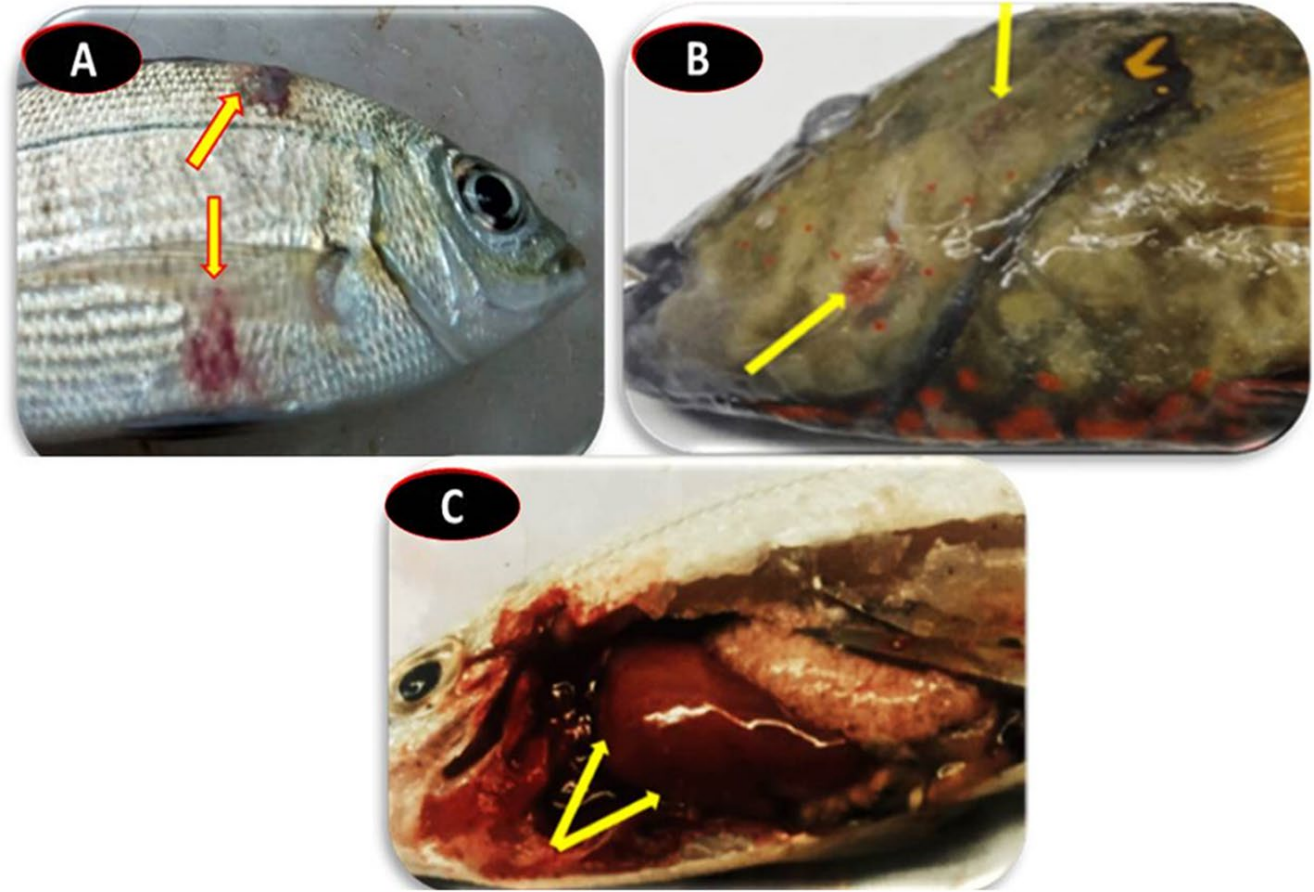
Clinical signs of *P. glacincola* infection of some red sea fishes: **A** *Rhabdosargus haffara* hemorrhagic body ulcer; **B** *Cheilinus lunulatus* hemorrhagic body ulcer; **C** *Rhabdosargus haffara* liver congestion

The infected marine fishes showed a series of clinical signs that included lethargy and sluggish movement, hemorrhages and ulcers on the body and on the operculum, occasional scale loss, and fin congestion and rot, especially on the tail fin (Fig. 1A and B). Furthermore, congestion of the liver, spleen, and kidney were observed during the postmortem examination (Fig. 1C).

### Prevalence of *P. glacincola* infection

The clinical examination of 180 Red Sea fish revealed a total prevalence of 6.7% of *P. glacincola* infection among all examined fish. Furthermore, the prevalence rates were 16.7%, 10%, and 13.3% among blackspot snapper (*Lutjanus ehrenbergii*), Haffara seabream (*Rhabdosargus haffara*), and broomtail wrasse (*Cheilinus lunulatus*) fish, respectively (Table 2).

**Table 2:** Occurrence of *P. glacincola* among six marine fish species of the Red Sea

Fish species		No. of examined fish	*Psychrobacter glacincola* infection	
**Scentific name**	**English name**		**No. of infected fish**	**Percentage of infected (%)**
		30	5	16.7
*Lutjanus ehrenbergii* (Peters, 1869)	Blackspot snapper	30	0	0
*Lethrinus borbonicus* (Valenciennes, 1830)	Snubnose emperor	30	0	0
*Siganus rivulatus* (Forsskål & Niebuhr, 1775)	Marbled spinefoot	30	3	10
*Rhabdosargus haffara* (Forsskål, 1775)	Haffara seabream	30	0	0
*Scarus ghobban* (Forsskål, 1775)	Blue-barred parrotfish	30	4	13.3
**Total**		**180**	**12**	**6.7**

### Bacteriological identification and morphological characteristics of the colonies

The colonies of these isolates were diplococci, cream-colored to unpigmented, smooth, and opaque with a buttery consistency after incubation at 22 °C for up to 48 h on tryptone soy agar. These microorganisms were Gram-negative non-motile diplococci and did not result in hemolysis of the blood agar, while they formed yellowish colonies on the MacConkey agar. Colonies also were able to grow at 1.5%, 3%, 7%, and 10% NaCl and at 4 °C, 22 °C, and 37 °C (supplemental file).

### Biochemical identification

As shown in Table 3, the 12 isolates were biochemically homogeneous and positive for cytochrome oxidase, catalase, and citrate utilization (CIT) and variable for urease (URE) and Voges Proskauer (VP) tests; all strains were negative for O-nitrophenyl-β-D-galactopyranoside (ONPG), arginine dihydrolase (ADH), lysine decarboxylase (LDC), ornithine decarboxylase (ODC), indole production (IND), H_2_S production (H_2_S), tryptophane deaminase (TDA), gelatin liquefaction tests, and acids from all sugars (glucose [GLU], mannitol [MAN], inositol [INO], sorbitol [SOR], rhamnose [RHA], sucrose [SUC], melibiose [MEL], amygdaline [AMY], and arabinose [ARA]).

**Table 3:** Biochemical characteristics of the recovered strain in the present study of *P. glacincola*

Test	Result	Test	Result
Oxidase test	+ ve	MacConky agar	+ ve
Catalase	+ ve	Blood haemolysis	-ve
Motility	+ ve	NaCl 1.5%	+ ve
Swarming	-ve	3%	+ ve
Growth at 4 °C	+ ve	7%	+ ve
37 °C		10%	+ ve
API20E tests			
Test	Result	Test	Result
ONPG	+ ve	GEL	-ve
ADH	-ve	GLU	-ve
LDC	-ve	MAN	-ve
ODC	-ve	INO	-ve
CIT	+ ve	SOR	-ve
H_2_S	-ve	RHA	-ve
URE	V	SAC	-ve
TDA	-ve	MEL	-ve
IND	-ve	AMY	-ve
VP	+ V	ARA	-ve

-ve: Negative + ve: positive V: variable

### Molecular identification

**Fig. 2:**
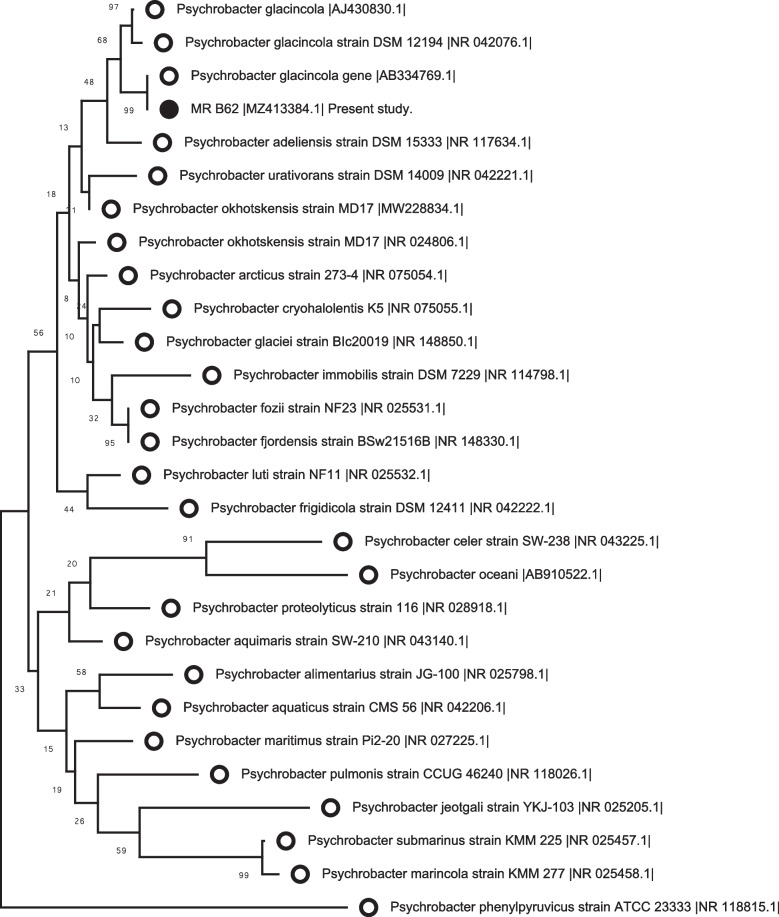
Neighbor-joining phylogenetic tree based on 16S rRNA gene sequences, showing the phylogenetic position of strain MRB63 and related members within the genus Psychrobacter, showing the evolutionary history. The optimal tree is shown. The percentage of replicate trees in which the associated taxa clustered together in the bootstrap test is shown next to the branches

The nucleotide sequences of the 16S rRNA gene could detect the isolates of bacteria at the species level according to levels of homology compared with the GenBank database. The recovered strain was identified and named MRB62. The draft genome sequence of strain MRB62 was deposited in National Center for Biotechnology Information (NCBI) and assigned an accession number (MZ413384.1). Interestingly, high similarity of the 16S rRNA gene sequences of the MRB62 isolate to that of *P. glacincola* T (Accession No. AB334769.1) with 100% identity is a remarkable finding. Furthermore, a closed genetic relationship was detected for the recovered strain (the strain MRB62 of the genus *Psychrobacter*) in the present study with some other strains in GenBank. Thus, an identity of 99.71%, 99.64%, 99.43%, and 98.86% was reported with that of *P. glacincola* LMG 21274 T (Accession No. AJ 430,830.1), *P. glacincola* DSM 12194 T (Accession No. NR 042,076.1), *P. adeliensis* DSM 15333 T (Accession No. 117634.1), and *P. immobilis* NBRCT 15,733 (Accession No. AJ NR113805.1), respectively. The matched sequences from GenBank were aligned using CLC Sequence viewer7 for similarity analysis. The phylogenetic tree was constructed using the MEGA 7 software (Fig. 2).

### Pathogenicity test

**Fig. 3:**
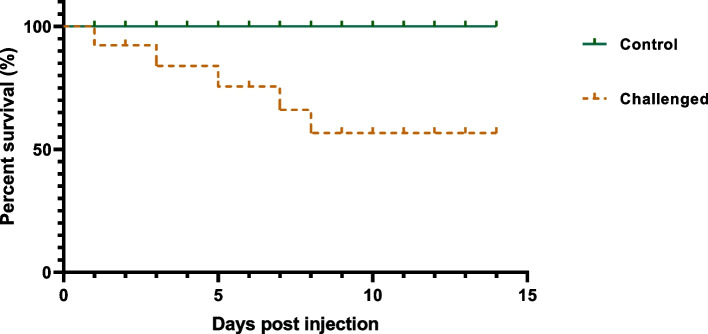
Kaplan–Meier survival curve of Haffara seabream (*Rhabdosargus haffara*) fish after challenged with *P. glacincola* AMP-FB1 isolate for 14 days; each fish was intraperitoneally injected with 0.1 mL of bacterial suspensions (3 × 10^7^ CFU/mL)

**Fig. 4:**
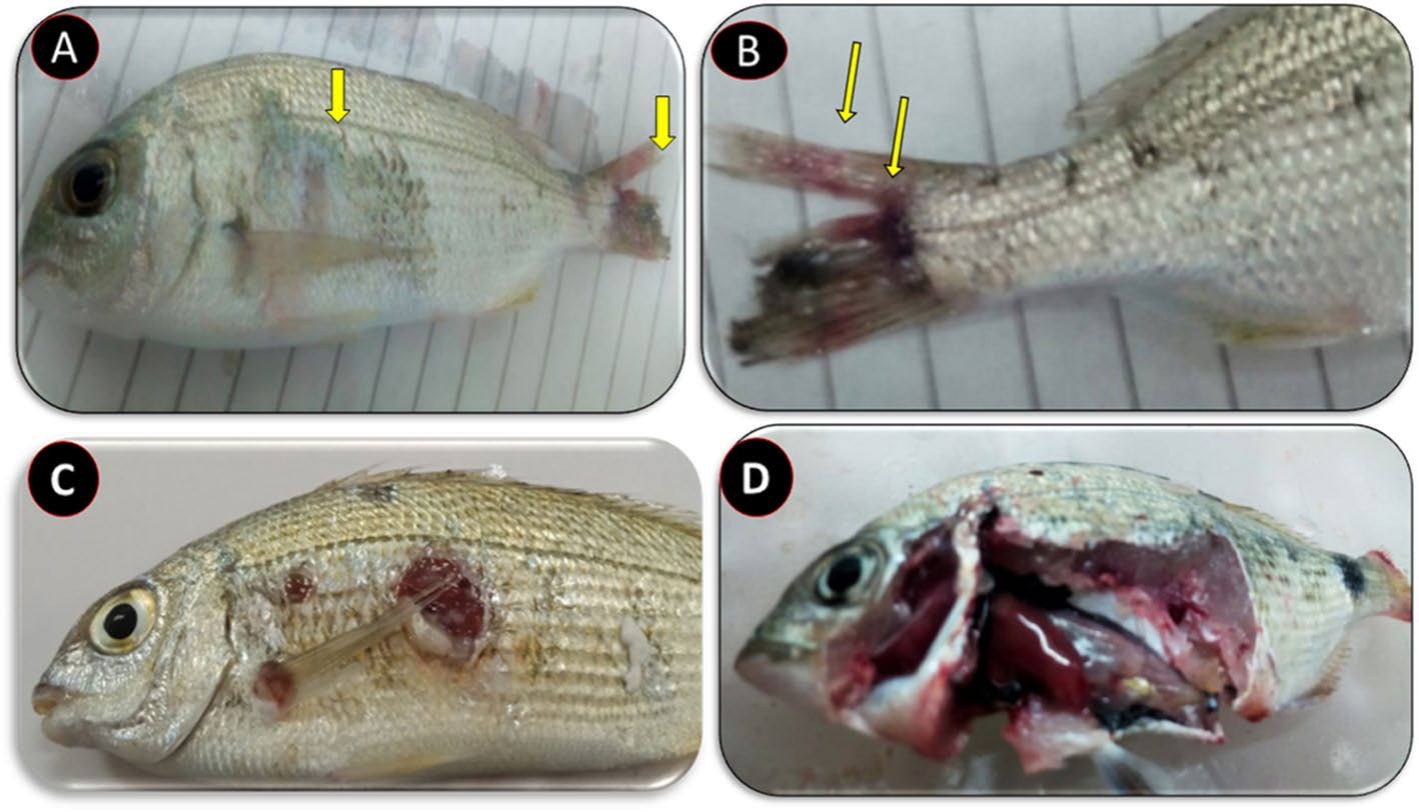
Clinical signs of Haffara seabream (*Rhabdosargus haffara*) experimentally challenge with *P. glacincola*, showing (**A**) scale loss and tail fin rot; **B** tail fin rot; **C** ulcer on the body; and (**D**) liver congestion

The *P. glacincola* experimentally infected Haffara seabream (*Rhabdosargus haffara*) showed a 23.3% mortality and clinical signs similar to those recorded in the naturally infected fish (Fig. 3). These clinical signs included skin hemorrhages, scale loss, tail fin rot and congestion, and liver congestion (Fig. 4). *P. glacincola* was also isolated and identified from the internal organs of the experimentally infected fish. The bacterial pathogen was re-isolated from the kidneys of infected fish.

### Antibiotic sensitivity test

In accordance with the antibiotic sensitivity test, the recovered isolates of *P. glacincola* were sensitive to amikacin, streptomycin, ciprofloxacin, gentamycin, chloramphenicol, tobramycin, and ofloxacin and resistant to tetracycline, cephalothin, cefotaxime, erythromycin, oxolinic acid, trimethoprim/sulfamethoxazole, clindamycin, flucloxacillin, and amoxicillin/clavulanic acid.

### Histopathological examinations

**Fig. 5:**
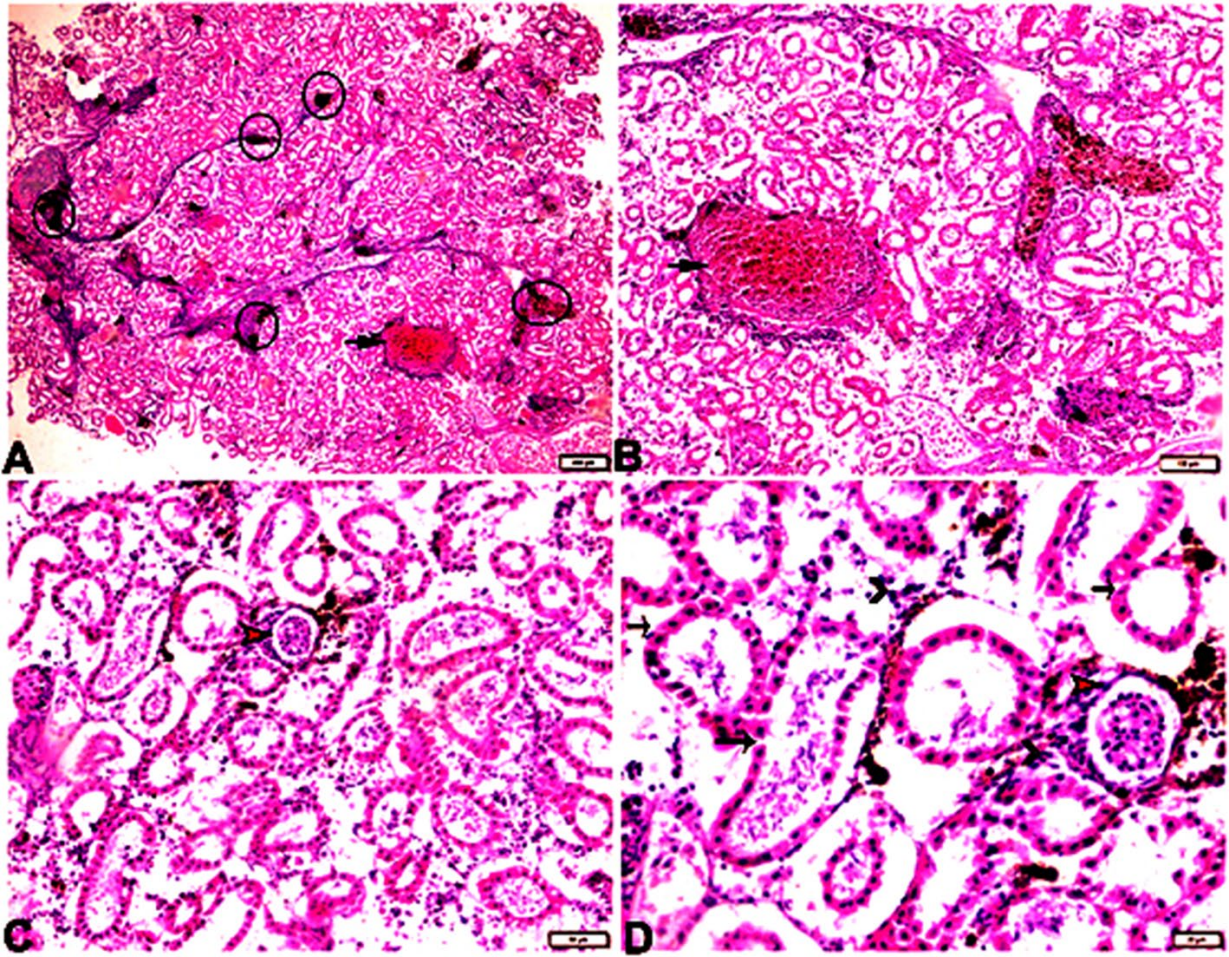
A photomicrograph of the posterior kidney sections from *Rhabdosargus haffara* fish experimentally infected with *Psychrobacter glacincola* shows congested vein, hyperplasia of the melanomacrophage centers, glomerular atrophy, necrosis and detachment of renal tubular epithelium, and interstitial mononuclear inflammatory cellular infiltration

**Fig. 6:**
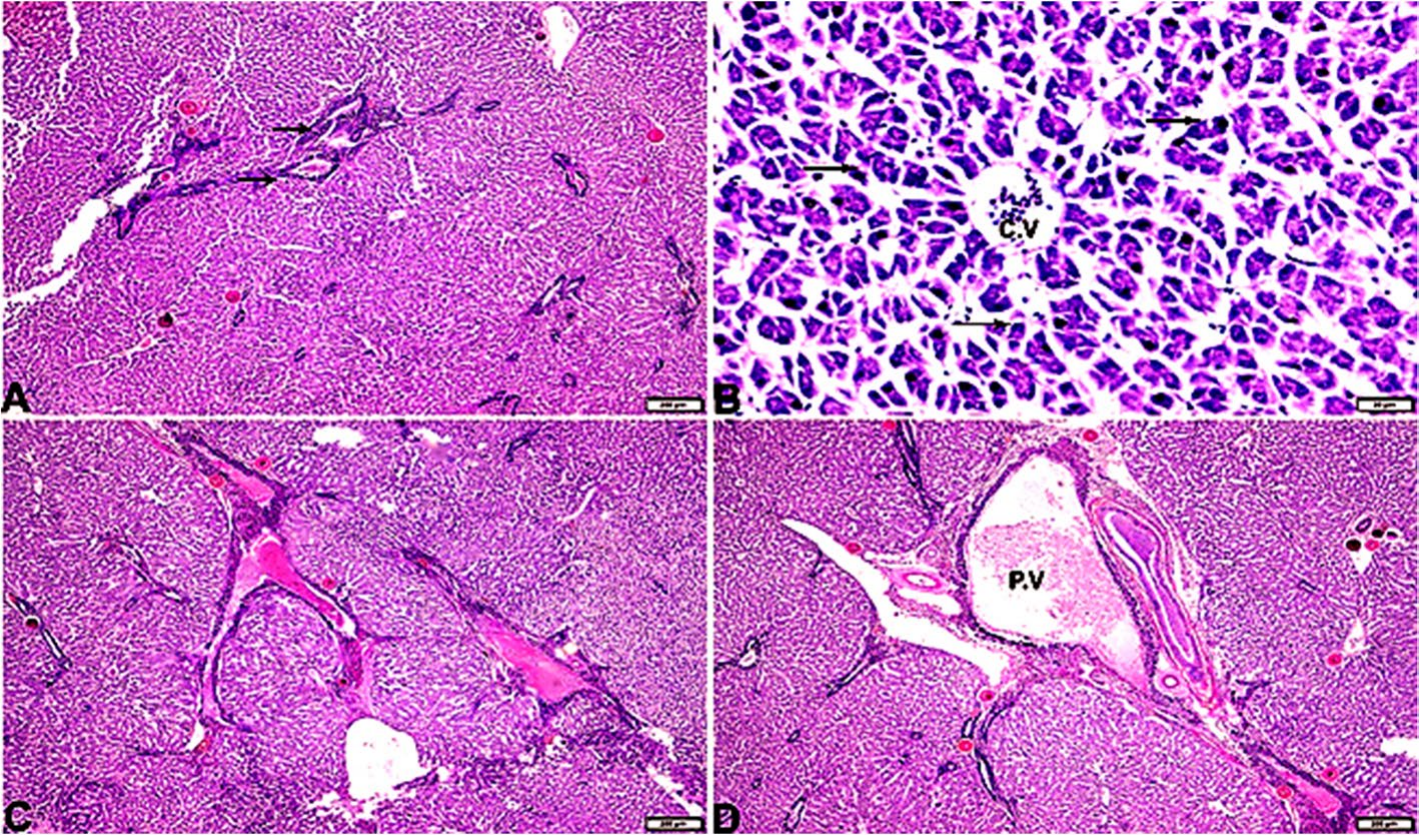
Photomicrograph of liver sections from *Rhabdosargus haffara* fish experimentally infected with *Psychrobacter glacincola* shows hepatic tissue showing necrosis and dissociation from its cord arrangement, congested veins, and dilated portal vein engorged with blood

A photomicrograph of the posterior kidney sections from *Rhabdosargus haffara* fish experimentally infected with *Psychrobacter glacincola* (Fig. 5) shows congested vein, hyperplasia of the melanomacrophage centers, glomerular atrophy, necrosis and detachment of renal tubular epithelium, and interstitial mononuclear inflammatory cellular infiltration. Photomicrograph of liver sections from *Rhabdosargus haffara* fish experimentally infected with Psychrobacter glacincola shows hepatic tissue showing necrosis and dissociation from its cord arrangement, congested veins, and dilated portal vein engorged with blood (Fig. 6).

## Discussion

In the Egyptian national income structure, aquaculture and fisheries represent an important sector [33]. However, this sector is challenged by a wide range of opportunistic pathogens that result in high mortalities and considerable economic losses. Bacterial pathogensare naturally present in the fish environment, and under some stressful conditions, they become the pathogens of the most important diseases in aquaculture [34]. Among others, *Psychrobacter* spp. could be considered as a potential bacterial pathogen in fish that results in high mortalities and considerable economic losses [15, 16]. Salinity levels in the Red Sea are higher than most saline water bodies in the world, which is between 36 and 38% [35]. *P. glacincola* is able to grow at up to 10% NaCl. Clearly, together with the investigation of the pathogen characteristics, providing updates and baseline information regarding the occurrence of *Psychrobacter* spp. infection among marine fishes is crucial for the implementation of the appropriate measures to prevent and control the infection. Reviewing the available literature, no previous study has reported elucidating the novel pathogenic strain of *P. glacincola* infection in fish either at a national or international level. However, *P. glacincola* as a novel strain was identified from sea ice cores in Antarctica [22], mud of the Shetland Islands [23], processed fresh edible sea urchin in Tokyo [24], and red tanner crab as reported by several previous studies [16]. Additionally, other previous studies [23, 36] registered *P. glacincola* in NCBI following isolation from marine environments and sea urchins. Through the isolation, identification, and characterization of the bacterium by bacteriological, biochemical, and molecular methods, the present work provides a novel contribution in relation to the occurrence of a novel pathogenic strain of *P. glacincola* in wild marine fishes in Egypt.

In the present work, the bacteriological examination of the examined fish revealed that the overall prevalence of *P. glacincola* infection among examined fish was 6.7%, while the individual prevalence rates were 16.7%, 10%, and 13.3% among *Lutjanus ehrenbergii, Rhabdosargus haffara,* and *Cheilinus lunulatus* fish, respectively. Conversely, *P. glacincola* could not be detected among *Lethrinus borbonicus, Siganus rivulatus,* and *Scarus ghobban*. Reviewing the available literature, no previous study has reported for *P. glacincola* infection among fishes worldwide, and the possible explanation for this prevalence may be attributed to the difference in susceptibility of fish species to the infection [37, 38].

In accordance with their clinical impact, the infected species showed several clinical signs and postmortem lesions as a result of *P. glacincola* infection. Blackspot snapper (*Lutjanus ehrenbergii*), Haffara seabream (*Rhabdosargus haffara*), and snubnose emperor (*Lethrinus borbonicus*) fishes of the Red Sea at Hurghada city showed lethargy and sluggish movement, hemorrhages and ulcers on the body and operculum, scale loss, fin congestion, and rot, especially in the tail fins, combined with congestion of the liver, spleen, and kidney.

It is noteworthy to state that there is no available literature that reported the clinical signs of *P. glacincola* infection. This study’s results corroborate to some extent with that of Hisar et al. [15], who found skin discoloration, gill paleness, abnormal swimming, and internal organ congestion in rainbow trout infected with other *Psychrobacter* spp. The *P. glacincola*-infected fishes showed frayed fins and fin rot that adversely affected the swimming activities and foraging behavior of the diseased fish, leading to loss of condition and weakness [39–41]. The diffuse hemorrhages on the fish body could be attributed to the secretion of some enzymes such as elastase and hemolysin that damage the blood vessels, leading to blood leakage [42]. Moreover, the clinical signs and postmortem lesions of the diseased fishes may be attributed to the extracellular products of *Psychrobacter* spp. such as proteases and hyaluronidase, which are involved in the development of clinical pathology and lesions [43, 44].

Isolation and identification of the causative agents remain one of the main interventions for infection control [45]. To identify and characterize bacterial pathogens, phenotyping is commonly used in combination with genotyping [45–47]. Likewise, biochemical characterization has been proven to be a valuable method for the typing and differentiation of several bacterial fish pathogens [48–50]. In this study, the phenotyping of the recovered isolates showed that the morphological characteristics of the colonies were cream-colored, unpigmented, smooth, and opaque with a buttery consistency. Additionally, yellowish colonies were formed on MacConkey agar with no hemolysis on blood agar. Biochemically, the isolates were homogeneous and positive for cytochrome oxidase, catalase, and citrate utilization, while negative for lysine decarboxylase, ornithine decarboxylase, arginine dihydrolase, indole production, H_2_S production, tryptophane deaminase, gelatin liquefaction test, and acid from all sugars. Our findings are in accordance with the results reported by Bowman et al. (1997) [22] and Garcia-Lopez et al. (2014) [51], who reported similar biochemical reactions with *P. glacincola*. Therefore, variations in any biochemical characteristics may be attributed to the presence or absence of plasmid(s) or mobile genetic elements that control its metabolic traits [52].

In accordance with the molecular methods, the phylogenetic analysis based on 16S rRNA gene sequence is an important tool, which confirms the genetic relatedness and stands alongside the biochemical tests and bacteriological tests for accurate and quick identification of bacteria [53–55]. The 16S rRNA gene sequence “alongside the biochemical tests” provides an accurate and rapid identification of the bacterial pathogen [4, 54, 56], and the phylogenetic analysis of 16S rRNA gene allows and confirms the identification of unknown bacterial isolates [57]. In this study, the phylogenetic analysis identified the recovered strain as *P. glacincola* (MRB62) based on the 16S rRNA gene sequence. Comparing the 16S rRNA gene sequence of the present strain (*P. glacincola* MR B62) with the known 16S rRNA gene sequences of *Psychrobacter* spp. on GenBank databases revealed a close similarity of 100% with *P. glacincola* T (Accession No. AB334769.1) [24], and the partial 16S ribosomal RNA gene sequence of this strain was deposited in NCBI and assigned accession number MZ413384.1.

Based on these results, this is the first study to report the occurrence of *P. glacincola* infection among snubnose emperor (*Lethrinus borbonicus*), Haffara seabream (*Rhabdosargus haffara*), and broomtail wrasse (*Cheilinus lunulatus*) marine fishes of the Red Sea at Hurghada City, Egypt. Furthermore, the recovered strain (*P. glacincola* MR B62) of the present study revealed an identity of 99.71%, 99.64%, 99.43%, and 99.07% with that of *P. glacincola* LMG 21274 T (Accession No. AJ 430,830.1) [22], *P. glacincola* DSM 12194 T (Accession No. NR 042,076.1) [22], *P. adeliensis* DSM 15333 T (Accession No. 117634.1) [58], and *P. immobilis* NBRCT 15,733 (Accession No. AJ NR113805.1) [59], respectively. Bacteria with an identity of more than 98.7% in the 16S rRNA gene sequence are considered to be the same species which confirms the hypothesis of the present study [60].

The pathogen was isolated and identified from the experimentally challenged Haffara seabream (*Rhabdosargus haffara*) fish to fulfill Koch's postulates in accordance with the results of the pathogenicity test. The present study proved that the present *P. glacincola* isolate was pathogenic to Haffara seabream and the challenged fish showed 23.3% mortality rates and exhibited clinical signs similar to those of the naturally infected fishes that included skin hemorrhages, scale loss, tail fin rot and congestion, and liver congestion. The recorded clinical signs may be attributed to the extracellular products such as cytotoxins, hemolysin, protease, collagenase, and hyaluronidase that were released during the infection [61, 62]. The present study also showed that *P. glacincola* isolates were sensitive to amikacin, streptomycin, ciprofloxacin, gentamycin, chloramphenicol, tobramycin, and ofloxacin and resistant to tetracycline, cephalothin, cefotaxime, erythromycin, oxolinic acid, trimethoprim/sulfamethoxazole, clindamycin, flucloxacillin, and amoxicillin/clavulanic acid. Some of our results concur with a previous study [5], where *P. glacincola* was sensitive to streptomycin and gentamycin and resistant to tetracycline and ampicillin. Generally, the high variations in the antibiotic sensitivity test results may be due to the dramatic antimicrobial resistance growth and the bacterial isolate variations.

## Conclusions

Given the above information, the present study reported for the first time a novel pathogenic bacterial isolate named *P. glacincola* from naturally diseased Snubnose emperor (*Lethrinus borbonicus*), Haffara seabream (*Rhabdosargus haffara*) and Broomtail wrasse (*Cheilinus lunulatus*) marine fishes. The isolated strains were identified by their morphological and biochemical characteristics. In addition, the phylogenetic analysis of the 16S rRNA gene sequence of the present study MR B62 isolate revealed 100% identity with *P. glacincola* T (Accession No. AB334769.1) the recovered strain, *P. glacincola*, was also pathogenic to Haffara seabream (*Rhabdosargus haffara*) and sensitive to amikacin, streptomycin, ciprofloxacin, gentamycin, chloramphenicol, tobramycin, and ofloxacin. The present data suggest large-scale surveys of *P. glacincola* infection in the fish sector in Egypt, which might be helpful for the implementation of effective control strategies for combating this infection.

### Supplementary Information

**Additional file 1.**

## Data Availability

All data generated or analyzed during this study are included in this manuscript and supplementary files.

## References

[1] Canton H. Food and Agriculture Organization of the United Nations—FAO. Routledge: In The Europa Directory of International Organizations; 2021. pp. 297-305.‏

[2] Liu PC, Lin JY, Hsiao PT, Lee KK (2004). Isolation and characterization of pathogenic Vibrio alginolyticus from diseased cobia Rachycentron canadum. J Basic Microbiol.

[3] Mohanty BR, Sahoo PK (2007). Edwardsiellosis in fish: a brief review. J Biosci.

[4] Srinivasan R, Karaoz U, Volegova M, MacKichan J, Kato-Maeda M, Miller S (2015). Use of 16S rRNA gene for identification of a broad range of clinically relevant bacterial pathogens. PLoS ONE.

[5] Romanenko LA, Schumann P, Rohde M, Lysenko AM, Mikhailov VV, Stackebrandt E (2002). Psychrobacter submarinus sp. nov. and Psychrobacter marincola sp. nov., psychrophilic halophiles from marine environments. Int J Syst Evol Microbiol.

[6] Rodrigues DF, da Jesus C, E, Pellizari VH, Gilichinsky D, Sepulveda-Torres L,  (2009). Biogeography of two cold-adapted genera: psychrobacter and Exiguobacterium. ISME J.

[7] Prabagaran SR, Manorama R, Delille D, Shivaji S (2007). Predominance of Roseobacter, Sulfitobacter, Glaciecola and Psychrobacter in seawater collected off Ushuaia, Argentina, Sub-Antarctica. FEMS Microbiol Ecol.

[8] Lo Giudice AL, Casella P, Caruso C, Mangano S, Bruni V, De Domenico M (2010). Occurrence and characterization of psychrotolerant hydrocarbon-oxidizing bacteria from surface seawater along the Victoria Land coast (Antarctica). Polar Biol.

[9] Kristiansen M, Ringo E (2013). Evaluation of prebiotic and probiotic effects on the intestinal gut microbiota and histology of atlantic salmon. International Aquafeed.

[10] Ringø E, Sperstad S, Myklebust R, Refstie S, Krogdahl Å (2006). Characterisation of the microbiota associated with intestine of Atlantic cod (Gadus morhua L.). Aquaculture.

[11] Steinum T, Sjåstad K, Falk K, Kvellestad A, Colquhoun DJ (2009). An RT PCR-DGGE survey of gill-associated bacteria in Norwegian seawater-reared Atlantic salmon suffering proliferative gill inflammation. Aquaculture.

[12] Svanevik CS, Lunestad BT (2011). Characterisation of the microbiota of Atlantic mackerel (Scomber scombrus). Int J Food Microbiol.

[13] Makled SO, Hamdan AM, El-Sayed A-FM, Hafez EE (2017). Evaluation of marine psychrophile, Psychrobacter namhaensis SO89, as a probiotic in Nile tilapia (Oreochromis niloticus) diets. Fish Shellfish Immunol.

[14] Sun Y-Z, YANG H-L, Ma R-L, ZHANG C-X, Lin W-Y,  (2011). Effect of dietary administration of Psychrobacter sp. on the growth, feed utilization, digestive enzymes and immune responses of grouper Epinephelus coioides. Aquacult Nutr.

[15] Hisar O, Yanik T, Hisar SA. Clinical And Pathological Investigation of *Psychrobacter Immobilis* Infection in Rainbow Trour (*Oncorhynchus Mykiss,* Walbaum). 2002.‏

[16] McCarthy Ú, Stagg H, Donald K, Garden A, Weir S (2013). Psychrobacter sp. isolated from the kidney of salmonids at a number of aquaculture sites in Scotland. Bull Eur Assoc Fish Pathol.

[17] Gini GA (1990). Ocular infection caused by Psychrobacter immobilis acquired in the hospital. J Clin Microbiol.

[18] Hudson MJ, Hollis DG, Weaver RE, Galvis CG (1987). Relationship of CDC group EO-2 and Psychrobacter immobilis. J Clin Microbiol.

[19] Leung WK, Chow VC, Chan MC, Ling JM, Sung JJ (2006). Psychrobacter bacteraemia in a cirrhotic patient after the consumption of raw geoduck clam. J Infect.

[20] Lloyd-Puryear M, Wallace D, Baldwin T, Hollis DG (1991). Meningitis caused by Psychrobacter immobilis in an infant. J Clin Microbiol.

[21] Lozano F, Florez C, Recio FJ, Gamboa F, Gómez-Mateas JM, Martín E (1994). Fatal Psychrobacter immobilis infection in a patient with AIDS. AIDS.

[22] Bowman JP, Nichols DS, McMEEKIN TA (1997). Psychrobacter glacincola sp. nov., a halotolerant, psychrophilic bacterium isolated from Antarctic sea ice. Syst Appl Microbiol.

[23] Bozal N, Montes MJ, Tudela E, Guinea J (2003). Characterization of several Psychrobacter strains isolated from Antarctic environments and description of Psychrobacter luti sp. nov. and Psychrobacter fozii sp. nov. Int J Syst Evol Microbiol.

[24] Kajikazawa T, Sugita T, Nishikawa A (2007). Comprehensive identification of bacteria in processed fresh edible sea urchin using 16S ribosomal DNA sequence analysis: the products contain various food poisoning-related bacteria and opportunistic bacterial pathogens. J Health Sci.

[25] Azevedo JS, Correia A, Henriques I (2013). Molecular analysis of the diversity of genus Psychrobacter present within a temperate estuary. FEMS Microbiol Ecol.

[26] Muñoz-Villagrán CM, Mendez KN, Cornejo F, Figueroa M, Undabarrena A, Morales EH (2018). Comparative genomic analysis of a new tellurite-resistant Psychrobacter strain isolated from the Antarctic Peninsula. PeerJ.

[27] Schäperclaus W (1992). Fish diseases.

[28] Noga EJ. Fish disease: diagnosis and treatment. Wiley; 2010.‏

[29] Frank JA, Reich CI, Sharma S, Weisbaum JS, Wilson BA, Olsen GJ (2008). Critical evaluation of two primers commonly used for amplification of bacterial 16S rRNA genes. Appl Environ Microbiol.

[30] Polz MF, Cavanaugh CM (1998). Bias in template-to-product ratios in multitemplate PCR. Appl Environ Microbiol.

[31] Kumar S, Stecher G, Tamura K (2016). MEGA7: molecular evolutionary genetics analysis version 7.0 for bigger datasets. Mol Biol Evol.

[32] Oh WT, Kim JH, Jun JW, Giri SS, Yun S, Kim HJ (2019). Genetic characterization and pathological analysis of a novel bacterial pathogen, Pseudomonas tructae, in rainbow trout (Oncorhynchus mykiss). Microorganisms.

[33] Abd El Tawab A, Ibrahim AM, Sittien A (2018). Phenotypic and Genotypic characterization of Vibrio species isolated from marine fishes. Benha Vet Med J.

[34] Olsson J, Jöborn A, Westerdahl A, Blomberg L, Kjelleberg S, Conway P (1998). Survival, persistence and proliferation of Vibrio anguillarum in juvenile turbot, Scophthalmus maximus(L.), intestine and faeces. J Fish Dis.

[35] Van Der Merwe R, Röthig T, Voolstra CR, Ochsenkühn MA, Lattemann S, Amy GL. High salinity tolerance of the Red Sea coral Fungia granulosa under desalination concentrate discharge conditions: an in situ photophysiology experiment. Frontiers in Marine Science. 2014;1:58.‏

[36] Panigrahi A, Kiron V, Satoh S, Hirono I, Kobayashi T, Sugita H (2007). Immune modulation and expression of cytokine genes in rainbow trout Oncorhynchus mykiss upon probiotic feeding. Dev Comp Immunol.

[37] Carol GR, Jeyasanta KI, Mani AE, Patterson J (2013). Prevalence of Pseudomonas sp in fin fishes and their antibiotic susceptibility. J Pure Appl Microbiol.

[38] LÓPEZ JR, et al. *Pseudomonas baetica*: pathogenicity for marine fish and development of protocols for rapid diagnosis. FEMS Microbiology Letters. 2017;364:3.‏ ‏ 10.1093/femsle/fnw286 28011698

[39] Khalil F, Emeash H (2018). Behavior and stereotypies of Nile tilapia (Oreochromis niloticus) in response to experimental infection with Aeromonas hydrophila. Aquat Sci Eng.

[40] Kujur P, Parganiha A (2013). Social interaction in fish: A brief review. Journal of Ravishankar University-B.

[41] Martins CI, Galhardo L, Noble C, Damsgård B, Spedicato MT, Zupa W (2012). Behavioural indicators of welfare in farmed fish. Fish Physiol Biochem.

[42] Zhang XH, Austin B (2005). Haemolysins in Vibrio species. J Appl Microbiol.

[43] Loperena L, Soria V, Varela H, Lupo S, Bergalli A, Guigou M (2012). Extracellular enzymes produced by microorganisms isolated from maritime Antarctica. World J Microbiol Biotechnol.

[44] Perfumo A, Freiherr von Sass GJ, Nordmann EL, Budisa N, Wagner D (2020). Discovery and characterization of a new cold-active protease from an extremophilic bacterium via comparative genome analysis and in vitro expression. Front Microbiol.

[45] Franco-Duarte R, Černáková L, Kadam S, Kaushik KS, Salehi B, Bevilacqua A (2019). Advances in chemical and biological methods to identify microorganisms-from past to present. Microorganisms.

[46] Coquet L, Cosette P, Quillet L, Petit F, Junter GA, Jouenne T (2002). Occurrence and phenotypic characterization of Yersinia ruckeri strains with biofilm-forming capacity in a rainbow trout farm. Appl Environ Microbiol.

[47] Bochner BR (2009). Global phenotypic characterization of bacteria. FEMS Microbiol Rev.

[48] Austin B, Austin DA, Blanch AR, Cerda M, Grimont F, Grimont PAD (1997). A comparison of methods for the typing of fish-pathogenic Vibrio spp. Syst Appl Microbiol.

[49] Austin B (2011). Taxonomy of bacterial fish pathogens. Vet Res.

[50] Fernández-Álvarez C, Santos Y (2018). Identification and typing of fish pathogenic species of the genus Tenacibaculum. Appl Microbiol Biotechnol.

[51] Garcia-Lopez ML, Santos JA, Otero A, Rodriguez-Calleja JM. Ch. Psychrobacter in: Encyclopedia of Food Microbiology 2 nd Edition, edited by Carl Batt and Mary Lou Tortorello (2014).‏

[52] Johnson DI. Bacterial virulence factors. In Bacterial pathogens and their virulence factors. Cham: Springer; 2018. pp. 1-38. ‏

[53] Young M, Artsatbanov V, Beller HR, Chandra G, Chater KF, Dover LG (2010). Genome sequence of the Fleming strain of Micrococcus luteus, a simple free-living actinobacterium. J Bacteriol.

[54] Buller NB. Bacteria from fish and other aquatic animals: A practical identification manual. CABI publishing; 2004.‏

[55] Tringe SG, Hugenholtz P (2008). A renaissance for the pioneering 16S rRNA gene. Curr Opin Microbiol.

[56] Clarridge JE (2004). Impact of 16S rRNA gene sequence analysis for identification of bacteria on clinical microbiology and infectious diseases. Clin Microbiol Rev.

[57] Ludwig W, Strunk O, Klugbauer S, Klugbauer N, Weizenegger M, Neumaier J (1998). Bacterial phylogeny based on comparative sequence analysis. Electrophoresis.

[58] Shivaji S, Reddy GS, Raghavan PU, Sarita NB, Delille D (2004). Psychrobacter salsus sp. nov. and Psychrobacter adeliensis sp. nov. isolated from fast ice from Adelie Land. Antarctica Syst Appl Microbiol.

[59] Juni E, Heym GA (1986). Psychrobacter immobilis gen. nov., sp. nov.: genospecies composed of gram-negative, aerobic, oxidase-positive coccobacilli. Int J Syst Evol Microbiol.

[60] Schleifer KH (2009). Classification of Bacteria and Archaea: past, present and future. Syst Appl Microbiol.

[61] Esteve C, Birbeck TH (2004). Secretion of haemolysins and proteases by Aeromonas hydrophila EO63: separation and characterization of the serine protease (caseinase) and the metalloprotease (elastase). J Appl Microbiol.

[62] Takahashi E, Ozaki H, Fujii Y, Kobayashi H, Yamanaka H, Arimoto S (2014). Properties of hemolysin and protease produced by Aeromonas trota. PLoS ONE.

