# Correction: Detection and description of a novel *Psychrobacter glacincola* infection in some Red Sea marine fishes in Hurghada, Egypt

**DOI:** 10.1186/s12917-023-03721-1

**Published:** 2023-09-15

**Authors:** Mohamed Raafat El‑Sayed, Arafah M. Emam, Ahmed Elsayed Osman, Mohamed Abd El‑Aziz Ahmed Abd El‑Galil, Haitham Helmy Sayed

**Affiliations:** 1 Department of Fish Diseases and Management, Faculty of Veterinary Medicine, Sohag University, Sohag, Egypt; 2 National Institute of Oceanography and Fisheries, NIOF, Cairo, Egypt; 3 Department of Biochemistry, Faculty of Veterinary Medicine, Sohag University, Sohag, Egypt; 4 Department of Microbiology, Faculty of Veterinary Medicine, Sohag University, Sohag, Egypt

**Correction**: ***BMC Vet Res*19, 23 (2023)**

10.1186/s12917-022-03542-8

Following publication of the original article [1], the authors identified an error in Fig. 5. The correct figure is given below.

**Fig. 5:**
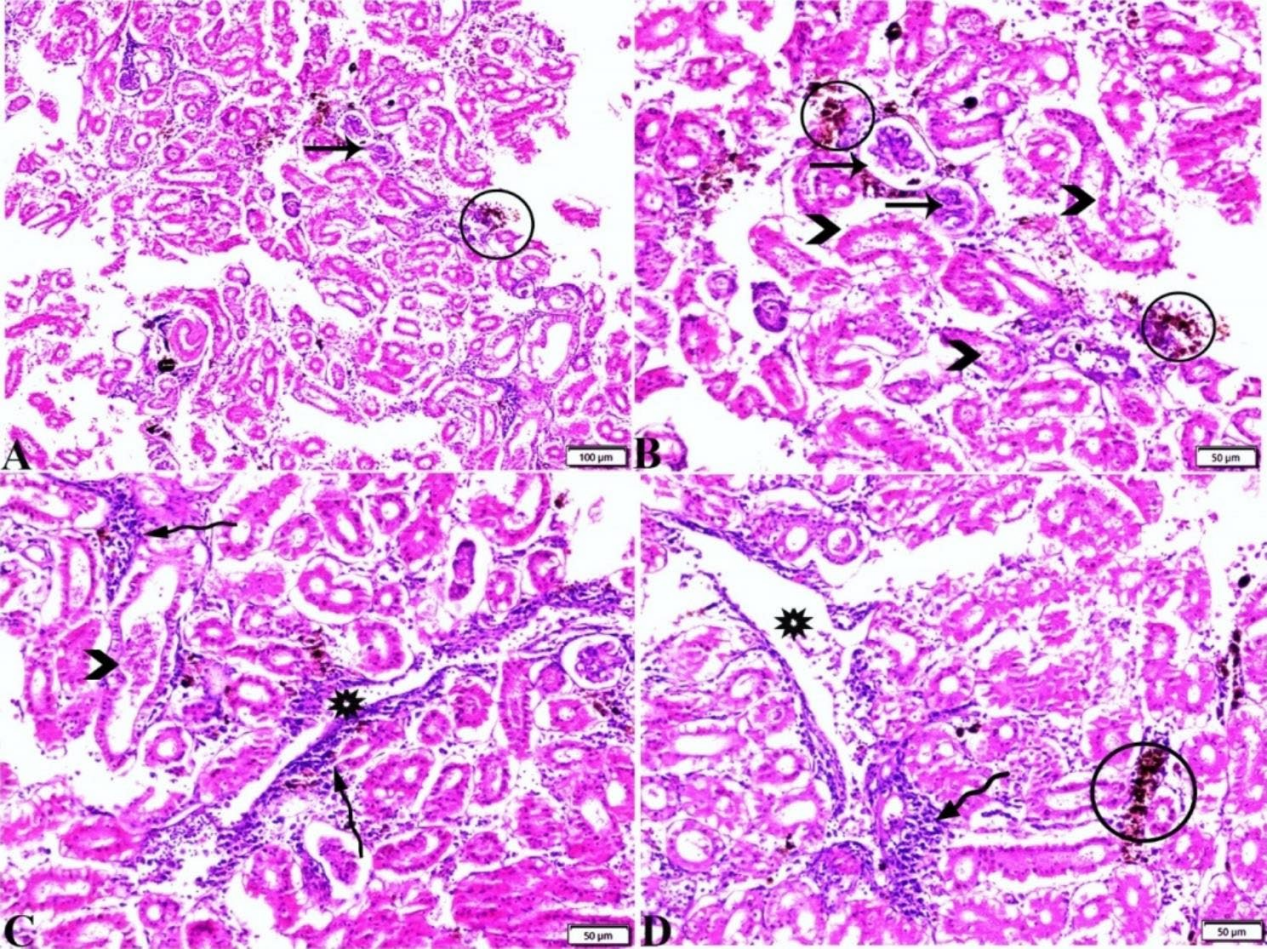
Photomicrograph (A magnified in B, C&D) of the posterior kidney sections from *Rhabdosargus haffara* infected with *psychrobacter glacincola* shows veins congestion (stars), melanomacrophage hyperplasia (circle), glomerular atrophy (arrows), renal tubular epithelium necrosis and detachment (arrowheads), and interstitial mononuclear inflammatory cellular infiltration. H&E stain. The bar size was indicated under pictures
