# Supplementary material for: Detection and description of a novel Psychrobacter glacincola infection in some Red Sea marine fishes in Hurghada, Egypt
Source: BMC Vet Res. 2023 Jan 30;19:23. doi: 10.1186/s12917-022-03542-8 (PMC9885648; doi:10.1186/s12917-022-03542-8)
Supplement: Supplementary file 1 — Additional file 1. [file 12917_2022_3542_MOESM1_ESM.docx]

| 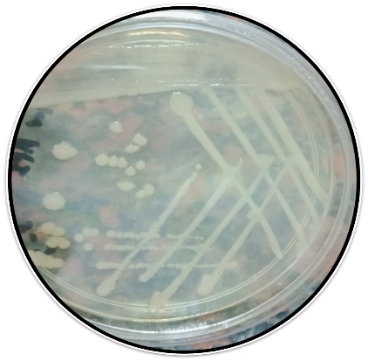 | |
| --- | --- |
| Photo (1): *Ps. glacincola* colonies on tryptone soya agar are cream-colored, unpigmented, smooth and opaque with a buttery consistency | |
| 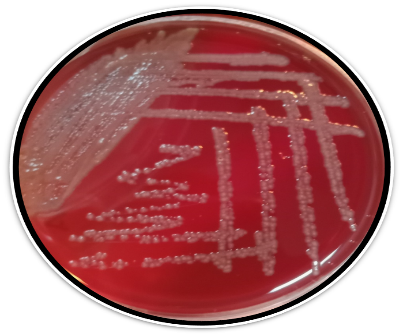 | 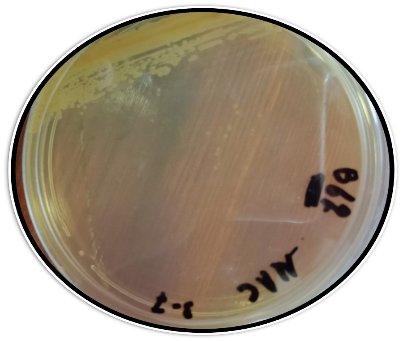 |
| Photo (2): *Ps. glacincola* colonies on blood agar without hemolysis | Photo (3): *Ps. glacincola* yellowish colonies on MacConkey agar |


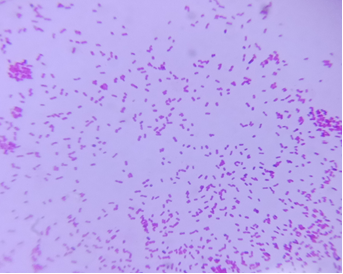


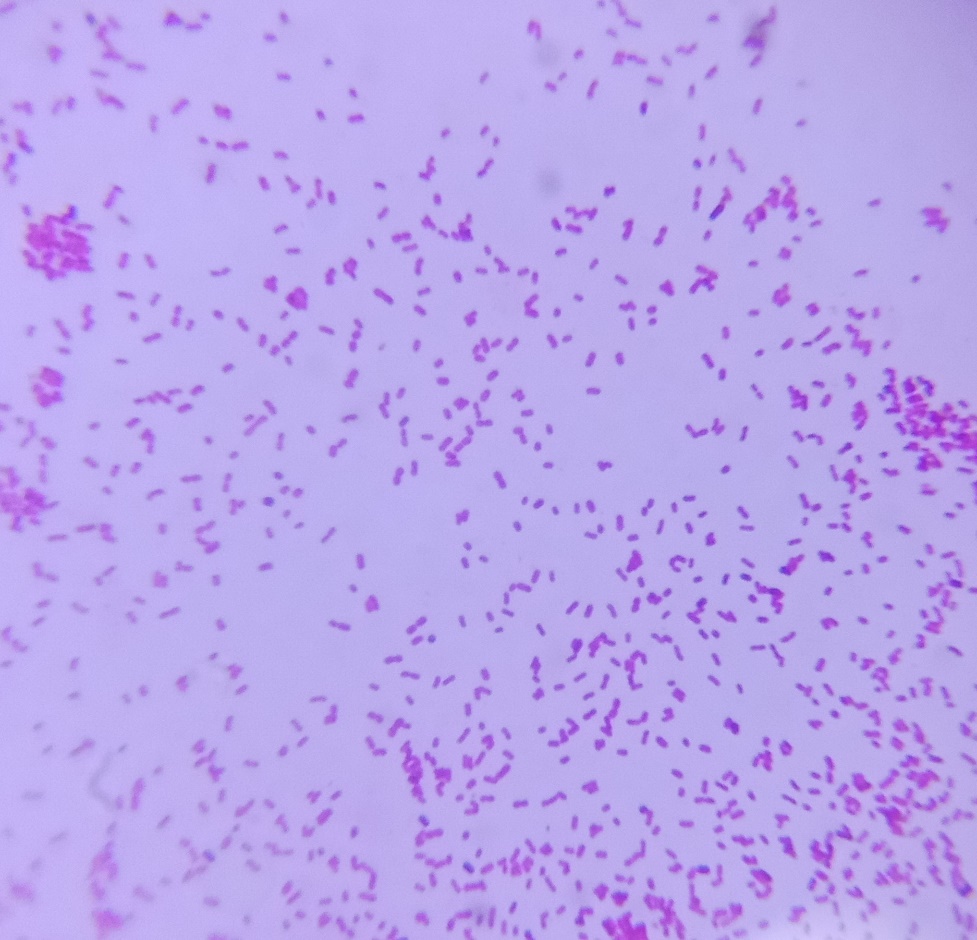


Microscopical examination for Gram**-**stained smears prepared from *Psychrobacter* suspected colonies, Gram negative coccobacilli which are often found as diploforms
